# Government (Teaching) and Municipal (Non-Teaching German Hospitals


**Published:** 1912-11-23

**Authors:** Henry Burdett


### GOVERNMENT (Teaching) and MUNICIPAL (Non-Teaching) GERMAN HOSPITALS.*

By SIR HENRY BURDETT, K.C.B.

### A German Critic on Voluntary Methods.

A distinguished professor of an English University, who has spent some months in a University Hospital in Germany under one of the ablest and most humane of German professors, when discussing with the writer the relative merits of voluntary and State hospitals, handed him a copy of the current week's *Punch* with his finger on p. iii. This page is headed "Hospitals and Charities," and he positively shuddered as he read out from the advertisements: "Help very earnestly solicited"; "In great need of funds"; "The Committee plead for donations and legacies." "Horrible!" he exclaimed, with his finger on the last advertisement. "The treatment of the sick is essential to the health and happiness of all classes of the community. Here you have a people rejoicing, and making a parade of the neglect of this vital question of adequate hospital provision throughout England. That is to say, they are not ashamed of these advertisements, and they rather pride themselves upon seeing them in print! I repeat, horrible. For to one possessed of the knowledge of the requirements of the residents in towns especially, it is amazing that in the twentieth century the existing hospital provision in the British Islands should be so inadequate and so much a matter of chance gifts by a few of the best of the race. You have just had a system of National Insurance set up, and here again your Parliament has sanctioned a measure which is unworkable as it stands. At any rate, do let us combine all our forces and teach Parliament and the people of these islands that health means adequate hospital provision, for adequate hospital provision means the maximum of earning power to the working classes and the maximum of comfort and happiness in the home." We pointed out that the adoption of the *pro rata* system in connection with the voluntary hospitals, with its financial clause making ready provision for all capital sums required to be expended on buildings, would remove the necessity for undignified appeals on behalf of voluntary hospitals, if, indeed, there has ever really been any necessity to issue them, or any justification either. Further, we beg leave to doubt the wisdom of such efforts, having regard to the results we have seen obtained from them, as compared with those derived from the issue of more businesslike, self-respecting, and dignified hospital literature.

It is well sometimes to see how our individual efforts in a good cause may be regarded by foreigners who look upon the voluntary system as a degradation, and not a glory, to any nation that adopts it. We mention this experience because at the moment all parties in the State seem agreed that it is of the first importance for the well-being of the patients, of the humbler classes especially, to strengthen the voluntary system and not to destroy it. Our Foreign friend was right from one point of view, in objecting to the advertisements to which he called attention. He properly maintained that a great hospital, when it wants money, should not "plead" and "beg" like a starveling for the money required to enable it to be efficient and up to date in the public service. A great and successful voluntary hospital, which is recognised as efficient in all its departments, should not "beg," but should claim from the public the money it requires for its work, and should base that demand upon accurate data recording the volume of the actual work successfully done, its variety and results. This criticism might be taken profitably to heart by those responsible for some advertisements published on behalf of certain voluntary hospitals. Many of them require editing, and it would not be a bad idea for these hospitals to take a leaf out of the book of the most up to date of our manufacturers, by combining together and securing the services of the best advertisement editor they can put their hands on. The better the literature, the greater the response. There is no limit to the telling points which a great hospital can make in the present day, providing they are marshalled by a practised hand, so as to attract and fix the attention of the reader. We may repeat that so judged the best of our voluntary hospitals, from the humanitarian's and the patient's point of view, must stand infinitely higher than any other type of hospital known to mankind in the present day.

### VI. German Practitioners' Fees from Hospital Patients.

We have been urging strongly in The Hospital during the last few weeks that the time has come for our hospital managers to awake and work to ensure the permanent financial strength and administrative excellence of the voluntary hospitals. Two points are regarded by some hospital managers as most difficult to determine: (1) The admission of patients who pay or are paid for to the extent of at least the cost entailed by their treatment in a hospital; and (2) the difficulty which might arise by the payment of fees to the practitioners who attend such cases in the hospitals. We showed quite clearly in The Hospital of October 26, pp. 93-4, that Germany has evolved a plan whereby every patient admitted to a hospital bed pays or is paid for at a fixed rate per diem during the whole time of his residence. Details of this simple system and its working could with equal readiness be applied to-morrow to every

* Previous articles of this series appeared in our issues of October 12, 19, 26, November 2 and 9.

voluntary hospital throughout the British Islands, with great advantage to the moral and material welfare of the patients and of the whole community. The system of *pro rata* payments for insured persons has commended itself with increasing acceptance to more and more voluntary hospitals, and to some highly placed in the Government, who take a deep and practical interest in the problem presented to voluntary hospital managers, by the passing of the National Insurance Act. This *pro rata* system preserves untouched the voluntary hospitals; it strengthens them; and it will give them the further advantage that the managers of every voluntary hospital which adopts it, once this system is put into force, can go to their supporters and say: "We have provided — beds, which we are devoting to the reception of that class of patient and their dependants whom this institution was originally founded to succour in the day of illness. The cost on an average of each patient during his or her residence in this hospital amounts to, say, from £3 15s. to £4 15s., so that every £1 15s. that you subscribe may pay the average cost of a week's hospital treatment for one such patient. By the plan we have now adopted we can guarantee you against all hospital abuse, and you may rest assured that the whole of the voluntary revenues of this institution will be devoted entirely to the class of people, only, who are entitled to receive, without direct payment, all the benefits which they need in the day of sickness or accident." We hope that the voluntary hospitals will forge ahead, by agreeing with the Government to take a loan for the sums they may require for hospital extension and new buildings for the reception of patients, on the plan set forth fully in The Hospital of October 26, 1912. They will then obtain the right to say to all classes: "We are now in a position to offer you and your family adequate hospital care at a reasonable rate, as and when you may require or desire it."

### Medical Directors and Professors.

Throughout Germany the system of working the hospitals, so far as medical treatment and the payment and fees of practitioners are concerned, is practically identical. Differences arise mainly owing to the size of the hospitals or to the rank of the medical directors of the surgical, medical, gynæcological, ophthalmic, and other special departments. Whether or not ambulance or polyclinic—that is, out-patients—are admitted to treatment may make some difference. In some of the best administered and larger hospitals the medical director and his *Oberarzt* or assistant director are permitted to see out-patient cases for a fee in rooms attached to his own bureau. Here it may be well to explain that in a large hospital of 1,500 or more beds there may be two medical or two surgical directors, each with assistants, and each having assigned to them a number of beds in separate pavilions. Where this system prevails the senior medical or surgical director is a member of the hospital executive which governs the institution; the second medical or surgical director is under the chief director, though the latter has nothing to do with the treatment of the cases of his colleague, except he may be called in consultation by him. In all matters of discipline, however, the senior is supreme, and his decision is final. The senior director and many of the assistants are professors attached to universities or teaching establishments, from which they receive handsome emoluments. Some of them in the larger cities, such as Berlin, Munich, and elsewhere, have consulting rooms and private patients, quite apart from the hospitals, but this is by no means a general rule, and as far as we could make out applies only to men of special eminence in the profession. The work of the senior and of the second director of a department and their assistants in one of the great German hospitals is full of responsibility, and entails arduous labour if the duties of the office are conscientiously discharged. A professorship in addition adds a good deal to the strain of hospital office, and when to these dual duties there be added the additional responsibility of treating private patients, the pressure upon the man is sometimes so heavy as to ensure his ultimate breakdown, unless he lightens the ship. Thus we saw one of the great practitioners of Berlin who was sacrificing himself by undertaking the threefold responsibilities just described. He was at the hospital early in the morning; he was lecturing in the course of the day; and he was seeing private patients until half-past eleven on the same evening, as we had actual evidence to prove. To be able to attain to a position where these threefold demands upon a professor's time are made means high qualifications and reputation. It entails continuous and unceasing toil in the earlier years of professional life, and a strain upon the practitioner himself which is so great as to be beyond the strength of ordinary men. We make bold to say that the few German hospitals where this system is permitted and prevails are in need of a strong administrator, who will come in and reorganise the work and the emoluments of the higher placed medical officials. Such an administrator should speedily secure that, the men of greatest eminence should obtain an adequate reward, without the necessity of undertaking the strain of labouring under a pressure of work which must ultimately undermine, if it does not actually break down, the vital energy of the strongest and healthiest of men.

### Hospital Medical Staff Salaries.

The salaries of the medical and surgical directors sometimes amount to as much as 20,000 marks a year each. In the municipal hospitals of Berlin these salaries vary from 7,800 to 5,000 marks for each director. These salaries are in addition to the dwelling, food, light, and heating accommodation, which is supplied free of cost in most of these hospitals to each member of the resident staff from the director downwards. In those cases where a director does not reside in the hospital a special grant is made in lieu of such perquisites, which grant varies from 1,500 to 2,000 marks a year according to rank and length of service. One surgical director in a smaller city receives 20,000 marks in salary; but even in such a case, in order presumably that he may be on the spot, it is usual to provide the director at least with an excellent suite of rooms within the hospital.

In Munich at the best hospital the yearly salaries are as follows: The surgical director receives 9,600 marks, with 2,600 marks allowance in lieu of a house and rations; the assistant director 5,460 marks, with apartments and board; the prosector 5,880 marks, without lodgment. Of the fifteen assistants, of whom thirteen are resident and two are not, the six surgical, six intern, and one Röntgen-ray assistant each receives a salary commencing at 1,200 marks and increasing to 2,000 marks in the fifth year, with lodgment and an allowance of 1,000 marks for food; the two pathologists, who are non-resident, are paid the same salary as the thirteen others. When this hospital is completed there will be an increase in the numbers of the senior and assistant resident medical staff.

At Dresden the medical directors in the public department receive 4,000 to 6,000 marks a year each; in the private patients' branch they receive from 3,000 to 4,000 marks; the prosector receives 4,000 to 5,000 marks; the chief practitioners, of whom there are two, receive 4,000 marks each, with an allowance of 1,050 marks for board, lodging, etc.; the resident medical assistants receive from 1,500 to 3,000 marks, with allowances varying from 1,300 to 2,500 marks; the chief dispenser gets from 4,500 to 5,000 marks, without allowances; the assistant dispensers get 2,800 marks, with rooms and food. Usually junior resident medical officers receive, in addition to their board and lodging, a salary of from 1,000 marks the first year to 1,300 marks the second. These salaries are increased to 2,400 marks in the case of lunatic asylums, and in the provincial lunatic asylums an assistant, after three years' service, may be promoted to the post of department medical officer, which entitles him to a salary of from 4,500 to 7,000 marks, with a residence, free board, fuel and light, etc. In some cases medical officers who offer their services are taken on to the resident staff and receive free board and, in addition, very occasionally 50 marks each per month. The senior members of the staff sometimes obtain consulting fees amounting to 30 marks. Sometimes all such fees ("opinions") are taken by the chief director, who may thus derive a not inconsiderable income.

### Fee Regulations, Out-Patients, and Operations.

The medical director, and sometimes his assistant, is permitted to take fees for the ambulance or polyclinic cases, and also fees for attendance upon the patients in the pay wards above the lowest class. These fees are regulated according to a scale which is fixed by the chief director of each hospital in the case of important operations or when occasion arises to deviate from the scale. A book is published which contains the Prussian Fee Regulations for Approved Doctors and Dentists, wherein are recorded long lists of operations of almost every kind from the slightest to the most important, with fees varying from 1 to 10 marks for such operations as simple bandaging, stitching and first bandage, removal of foreign bodies from natural openings, and the splinters of bones from a gun-wound. The cost of removing a tumour varies from 3 to 15 marks when it is located externally, to 200 marks when it is complicated; the charge is from 2 to 10 marks for setting fingers or toes, 15 to 30 marks for fractures of the thigh, 20 to 50 marks for those of the femur, with a proviso that in compound fractures these fees may be increased by from 10 to 50 marks. It costs from 20 to 150 marks to remove a foot or a hand; from 30 to 200 marks to amputate an arm or a leg; from 10 to 30 marks to remove wholly or partially a toe or a finger. Resections of various kinds cost from 30 to 100 marks; minor abdominal operations cost from 50 to 500 marks; the fee for lithotomy from 60 to 500 marks; whilst the charge for transfusion is only 30 to 60 marks. We have made these selections almost at random from some pages of printed matter, but they sufficiently indicate the range of fees authorised. These fees, it must be borne in mind, are the minimum and not the maximum charges for the operations in question.

Reverting next to the charges made for the general medical and surgical treatment of patients above the lowest class in the pay wards of general hospitals, the practice is to add the doctor's fee to the fee charged each patient per diem for residence. These fees are pretty uniform, and seem to run from 1s. 6d. to 2s. 6d. a day, that is, from half a guinea to 17s. 6d. per week, apart from the fees for operation, which are matters of private arrangement between the patient's friends and the practitioner, as settled by the chief director of these hospitals. In clinics and sanatoria the practitioner's fee for the highest class of patients varies from five to ten marks a day, and in the second class from three to five marks a day, and here the fees may be reduced in the case of patients whose stay is prolonged. In the case of out-patients the fees which prevail vary from nothing to 6d. per patient, put in a box, up to 2.50 marks, and higher fees according to the standing of the medical officer who attends to the patient. The hospitals have a curious regulation by which the prices charged to patients other than the lowest class are increased in some months and reduced in others. There is a further practice of increasing by 25 per cent. the charges made to patients who are strangers—that is, non-residents in the towns where the hospital is situated. In some municipal hospitals it was the practice to treat out-patients without charge; during recent years, as for example at the Charlottenburg Hospital since July 1911, patients who attend as out-patients must pay a fee which exceeds the lowest rate provided in the Fee Regulations for Approved Doctors. Members of approved societies who are *Kassen* patients pay 2.75 marks for each medical attendance, and four marke for each home visit. Labour cases pay ten marks, but the destitute pay no fee. In some polyclinics the fees charged are from one to two marks only. The plan is simplicity itself, for all the money is paid to the hospital cashier, and then credited to the particular member of the medical staff who has earned a fee by his treatment of the patient.

